# Mixed Systems of Quaternary Ammonium Foam Drainage Agent with Carbon Quantum Dots and Silica Nanoparticles for Improved Gas Field Performance

**DOI:** 10.3390/nano14191590

**Published:** 2024-10-01

**Authors:** Yongqiang Sun, Yongping Zhang, Anqi Wei, Xin Shan, Qingwang Liu, Zhenzhong Fan, Ao Sun, Lin Zhu, Lingjin Kong

**Affiliations:** 1 Petroleum Engineering College, Northeast Petroleum University, Daqing 163000, China; 13206816336@163.com (Y.S.); liuqingwang@163.com (Q.L.); fanzhenzhong@163.com (Z.F.); sunaonepu@hotmail.com (A.S.);; 2 The Fourth Oil Extraction Plant of Daqing Oilfield Co., Ltd., Daqing 163000, China; wei437447553@163.com (A.W.); konglingjin1234@163.com (L.K.); 3 Oil Production Engineering Research Institute of Daqing Oilfield Co., Ltd., Daqing 163000, China; 4 Key Laboratory of Marine Geology and Metallogeny, First Institute of Oceanography, Ministry of Natural Resources, Qingdao 266061, China; xshan@fio.org.cn

**Keywords:** foam drainage agent, silica nanoparticles, carbon quantum dots, carbon quantum dots/silica nanoparticles, foam drainage system, gas field

## Abstract

Foam drainage agents enhance gas production by removing wellbore liquids. However, due to the ultra-high salinity environments of the Hechuan gas field (salinity up to 32.5 × 10^4^ mg/L), no foam drainage agent is suitable for this gas field. To address this challenge, we developed a novel nanocomposite foam drainage system composed of quaternary ammonium and two types of nanoparticles. This work describes the design and synthesis of a quaternary ammonium foam drainage agent and nano-engineered stabilizers. Nonylphenol polyoxyethylene ether sulfosuccinate quaternary ammonium foam drainage agent was synthesized using maleic anhydride, sodium chloroacetate, N,N-dimethylpropylenediamine, etc., as precursors. We employed the Stöber method to create hydrophobic silica nanoparticles. Carbon quantum dots were then prepared and functionalized with dodecylamine. Finally, carbon quantum dots were incorporated into the mesopores of silica nanoparticles to enhance stability. Through optimization, the best performance was achieved with a (quaternary ammonium foam drainage agents)–(carbon quantum dots/silica nanoparticles) ratio of 5:1 and a total dosage of 1.1%. Under harsh conditions (salinity 35 × 10^4^ mg/L, condensate oil 250 cm^3^/m^3^, temperature 80 °C), the system exhibited excellent stability with an initial foam height of 160 mm, remaining at 110 mm after 5 min. Additionally, it displayed good liquid-carrying capacity (160 mL), low surface tension (27.91 mN/m), and a long half-life (659 s). These results suggest the effectiveness of nanoparticle-enhanced foam drainage systems in overcoming high-salinity challenges. Previous foam drainage agents typically exhibited a salinity resistance of no more than 25 × 10^4^ mg/L. In contrast, this innovative system demonstrates a superior salinity tolerance of up to 35 × 10^4^ mg/L, addressing a significant gap in available agents for high-salinity gas fields. This paves the way for future development of advanced foam systems for gas well applications with high salinity.

## 1. Introduction

Global economic growth has led to increasing demand for oil and natural gas [1]. In the latter stages of gas field development, production of gas drops to the critical liquid-carrying flow rate as the bottom hole pressure decreases. This liquid accumulation reduces gas production and can even halt it entirely [2], a process known as gas well flooding [3]. Additionally, condensate oil can obstruct reservoir pathways, further diminishing well productivity [4]. To address these challenges, various methods have been employed, including tubing, gas lift, and foam drainage [5]. The latter has emerged as a preferred approach, which involves the injection of foam drainage agents to reduce liquid buildup [6]. These agents decrease liquid surface tension, creating a low-density foam–liquid mixture that can be lifted by reservoir energy [7]. Foam drainage agents have been widely adopted globally to mitigate liquid accumulation in gas wells [8]. Successful applications include Bowman’s European operations [9], where gas production increased fivefold, and the Alliance shale gas field in Texas [10], where foam drainage significantly boosted recovery rates in over one third of production wells. A key advantage of foam drainage is its compatibility with existing downhole equipment.

The widespread application of foam drainage agents has highlighted their susceptibility to the harsh conditions often encountered in reservoirs, including high temperature, salinity, and condensate oil content [11]. Conventional foam drainage agents frequently prove inadequate in such environments. To address this challenge, the development of novel foam drainage agents through surfactant formulation has become a primary focus. Zhao [12] investigated a foam system composed of alkyl hydroxysulfonate betaine, dodecanol, and quaternary ammonium salt surfactants. This combination demonstrated synergistic effects, maintaining performance under conditions of 60 °C and 16 × 10^4^ mg/L salinity. Hui [13] synthesized a novel betaine Gemini surfactant, B18-4-18, exhibiting exceptional resistance to extreme reservoir conditions. Liquid-carrying capacity tests revealed strong performance: 92% in water at 258 °C, 84% in 150,000 mg/L saline water, and 67% in the presence of 10% condensate oil. These results underscore the potential of B18-4-18 as a promising foam drainage agent for challenging reservoir environments.

Previous studies have shown the efficacy of polymer-enhanced foam drainage agents in gas wells characterized by high salinity and oil content. Increased polymer concentration correlates with improved foam stability and overall agent performance [14]. Xu [15] proposed a novel ternary polymer, AVS, composed of AM, AMPS, and a functional monomer. This polymer exhibited exceptional foam stabilization under high salinity and temperature conditions. Building upon these findings, the impact of polyacrylamide (PAM) concentration and the degree of hydrolysis of partially hydrolyzed polyacrylamide (HPAM) on sodium dodecyl sulfate (SDS) foam stability has been investigated. Wu [16] employed molecular dynamics simulations to elucidate the underlying mechanisms of polymer–surfactant interactions in PAM-SDS and HPAM-SDS systems. Results indicate that elevated PAM concentration strengthens PAM-SDS interactions, enhancing foam stability. Furthermore, HPAM with a hydrolysis degree between 20% and 30% yielded the most stable foam within the HPAM-SDS system.

Nanotechnology, which involves the manipulation of matter at the nanoscale, has found widespread applications in energy, materials, and other domains [17,18,19]. In recent years, nano-stabilized foam technology has emerged as a promising approach for gas recovery [20,21]. By adsorbing to the gas–liquid interface, nanoparticles reinforce foam drainage agent action, creating a rigid framework that inhibits bubble coalescence and disproportionation. This leads to significantly enhanced foam stability compared to conventional foam drainage agents [22]. Worthen [23] demonstrated the synergistic effects of silica nanoparticles and octenyl amide propyl betaine in producing highly stable foam under demanding conditions of 19 MPa and 60 °C. Latif [24] further explored the synergistic interaction between surfactants and silica under high-salinity conditions, resulting in improved foam stability, film thickness, apparent viscosity, and foam volume. These studies collectively underscore the potential of nano-silica in augmenting foam stability.

The Hechuan gas field suffers from severe liquid accumulation, significantly curtailing gas production. Implementing foam drainage gas recovery is imperative to address this issue. The field’s challenging conditions include a temperature of 80 °C, a salinity of 325,000 mg/L, and high concentrations of calcium and magnesium ions. These extreme parameters render conventional foam drainage agents ineffective. High salinity demands specific surfactant properties [25,26,27]. Sun [28] employed molecular dynamics simulations to elucidate the impact of salt on foam stability and surfactant synergy at the gas–water interface. Condensate oil, primarily composed of low-molecular-weight hydrocarbons, exacerbates the problem due to its poor hydrophilicity [29,30,31,32,33]. Kofi [34] demonstrated the detrimental effect of oil on foam stability, highlighting the need for oil-tolerant formulations. To address these challenges, we developed a novel foam drainage agent incorporating sulfonic group surfactants for high-temperature resistance and zwitterionic surfactants for salinity tolerance [35,36,37,38]. To enhance condensate oil resistance, we integrated nanoparticles. Among these, carbon quantum dots (CQDs) and Nano-SiO_2_ particles have garnered significant attention [39,40]. Mesoporous Nano-SiO_2_ offer unique advantages due to their high specific surface area and pore structure [41,42,43]. We synthesized amorphous Nano-SiO_2_ particles using the Stöber method and hydrophobic modified them. CQDs were prepared from citric acid with dodecylamine grafting. Subsequently, mesoporous Nano-SiO_2_ and CQDs were combined to form CQDs/Nano-SiO_2_ composites. A nanoparticle foam drainage agent system was established and its performance evaluated through compounding experiments.

## 2. Materials and Methods

### 2.1. Materials

This study utilized Alkylphenol polyoxyethylene ether OP-14, sodium bisulfite, N,N-dimethylpropylenediamine, sodium chloroacetate, maleic anhydride, (3-chloropropyl)trimethoxysilane, ammonia water, tetraethyl orthosilicate (TEOS), palmitic acid, myristic acid, sodium hydroxide, hydrochloric acid, 3-dimethylaminopropylamine, citric acid, dodecylamine, fatty alcohol polyoxyethylene ether sulfate (AES), diesel, ethanol, water, calcium chloride, sodium chloride, potassium chloride, magnesium chloride, methanol, ethylene glycol, a Hechuan gas field gas well water sample, and Hechuan gas field condensate oil samples.

### 2.2. Methods

A Ross–Miles foam meter was employed for foam heights and half-life measurements. A fully automatic tensiometer was used to measure surface tension.

#### 2.2.1. Synthesis of Foam Drainage Agent

##### Selection of Molecular Structure for Foam Drainage Agent

To enhance foam drainage agent performance, surfactants incorporating sulfonic acid groups for thermal stability and amphoteric structures for mineralization resistance were selected. Given the gas field’s high mineralization (>250,000 mg/L), condensate oil content (>100 cm^3^/m^3^ natural gas), and water quality characteristics, foam drainage agent was designed with a molecular main chain carbon number of 14–16 and functional groups including carbonyl, quaternary ammonium, hydroxyl, polyoxyethylene, carboxyl, and sulfo groups to ensure compatibility and effectiveness under these challenging conditions.

Foam drainage agents were designed with a molecular backbone of 14–16 carbon atoms and incorporated functional groups, including carbonyl, quaternary ammonium, hydroxyl, polyoxyethylene, carboxyl, and sulfonic acid moieties. This molecular architecture was selected to ensure compatibility with the gas field’s specific conditions, characterized by high mineralization levels exceeding 250,000 mg/L, condensate oil content surpassing 100 cm^3^/m^3^ natural gas, and unique water quality characteristics. Foam drainage agent with a 14–16 carbon chain backbone exhibited favorable surface activity and wettability, readily adsorbing at the gas–liquid interface. Incorporation of hydroxyl and polyoxyethylene groups enhanced surface activity, hydrophilicity, and hard water tolerance. These hydrophilic moieties facilitated hydrogen bond formation with water, thickening the gas–liquid interface film, prolonging liquid drainage time, and ultimately improving foam stability and liquid-carrying capacity. The introduction of sulfonic acid groups augmented temperature and salt resistance by creating a hydrated interfacial film. Quaternary ammonium cations further improved water adaptability and salt tolerance.

By strategically incorporating sulfonic acid, quaternary ammonium, hydroxyl, polyoxyethylene, and carboxyl functional groups into a 14–16 carbon chain backbone, novel foam drainage agent was synthesized to effectively resist high mineralization (exceeding 250,000 mg/L) and condensate oil contamination (greater than 100 cm^3^/m^3^ natural gas) prevalent in the gas field environment.

##### Synthesis Process of Foam Drainage Agent

**Figure 1:**
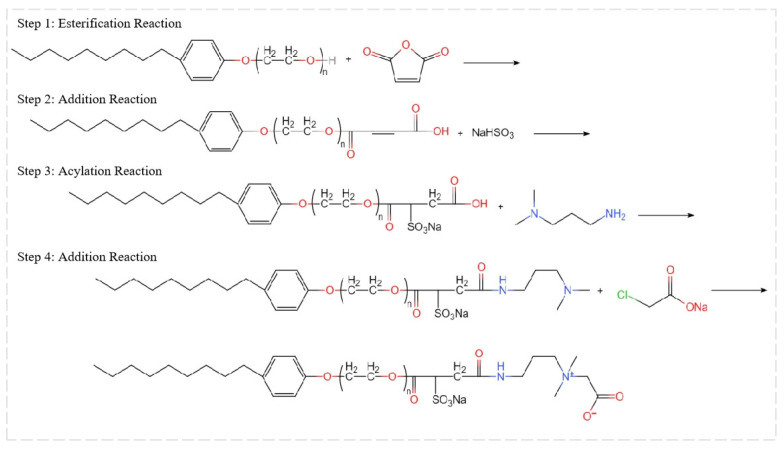
Reaction principle of the main foam drainage agent.

Nonylphenol polyoxyethylene ether sulfosuccinate quaternary ammonium salt (abbreviated as “QAFDA”; the following will use the abbreviation), an amphoteric surfactant containing anionic, cationic, and nonionic moieties, was synthesized using maleic anhydride, sodium chloroacetate, N,N-dimethylpropylenediamine, OP-14, and sodium bisulfite as precursors. Figure 1 outlines the four-step reaction pathway for the primary foam drainage agent, namely esterification reaction, addition reaction, acylation reaction, and addition reaction.

Step 1 (Esterification Reaction): The esterification reaction was initiated by adding a certain amount of Alkylphenol polyoxyethylene ether OP-14 to a dried 250 mL three-neck flask. Upon heating to a predetermined temperature, maleic anhydride was gradually introduced. The reaction mixture was continuously stirred within a constant-temperature water bath for a defined duration. Periodic sampling and acid value titration using a standardized NaOH solution monitored reaction progress. The reaction was terminated when the acid value stabilized, followed by the removal of unreacted maleic anhydride under reduced pressure.

Step 2 (Addition Reaction): The sulfonation reaction commenced by adjusting the pH with 30% NaOH aqueous solution and adding an equal amount of 30% NaHSO_3_ aqueous solution dropwise to the reaction flask. The mixture was stirred and heated under reflux in a constant temperature water bath for a specified period.

Step 3 (Acylation Reaction): In the subsequent acylation step, 4.81 g of N,N-dimethylpropylenediamine was added dropwise to the reaction mixture, followed by refluxing with condensation. The final product was obtained through evaporation under normal pressure.

Step 4 (Addition Reaction): The quaternization reaction was initiated by dissolving sodium chloroacetate in a specific water–ethanol mixture within a three-neck flask. The solution was heated to 85 °C for a reaction time of 9 h. Subsequent removal of ethanol and water was achieved through reduced pressure distillation. The crude product was filtered to eliminate excess inorganic salts and finally dried to yield the target foam drainage agent, nonylphenol polyoxyethylene ether sulfosuccinate quaternary ammonium salt.

#### 2.2.2. Synthesis of Foam Stabilizers

To enhance foam stability in the presence of condensate oil, nanoparticles were incorporated to reduce the system’s overall surface energy.

##### Synthesis of Silica Nanoparticles via the Stöber Method

Silica nanoparticles (abbreviated as “QAFDA”; the following will use the abbreviation) were synthesized using the Stöber method [44]. A mixture of 8 mL anhydrous ethanol, 12.5 mL distilled water, and 4.5 mL of ammonia solution was prepared and stirred at 25 °C and 350 rpm in a three-neck flask. Subsequently, a solution of 2.25 mL TEOS in 22.25 mL of anhydrous ethanol was rapidly added while maintaining vigorous stirring (1200 rpm) to prevent contact with the flask walls. The stirring speed was then reduced to 350 rpm, and the reaction continued at 25 °C for 2 h. The resulting product was centrifuged at 12,500 rpm, washed with distilled water (3–4 times), dried, and ground to yield white, powdery nano-SiO_2_ particles. To investigate the influence of ammonia concentration on particle size, the amount of ammonia solution was varied in subsequent experiments.

##### Synthesis of Modified Nano-SiO_2_ Particles

**Figure 2:**
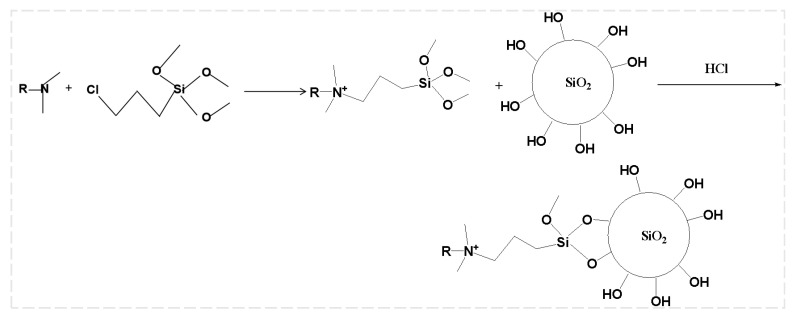
Reaction principle of silane quaternary ammonium-salt-modified SiO_2_.

Hydrophilic Nano-SiO_2_ particles possess a high density of hydroxyl groups on their surface, imparting strong hydrophilicity in aqueous environments. The reaction mechanism for this surface modification process is illustrated in Figure 2. To impart hydrophobicity, these hydroxyl groups were replaced with hydrophobic groups through surface modification using silane coupling agents [45]. Specifically, 3-Chloropropyltrimethoxysilane (CTS) was reacted with tertiary amines to produce silane quaternary ammonium salts containing a tetradecyl structure. These salts were subsequently used to modify the Nano-SiO_2_ particles, yielding hydrophobic Nano-SiO_2_ particles with a 3-(trimethoxysilyl)propyl dimethyl tetradecylammonium chloride coating [46]. 

The foam drainage agent was synthesized through a three-step process. In the first step, lauric acid and N,N’-dimethyl-1,3-propanediamine were reacted under a nitrogen atmosphere to produce a tertiary amine. The reaction was carried out in a three-necked flask at 110 °C, with the amine added dropwise. The temperature was then increased to 145 °C to complete the reaction.

In the second step, the tertiary amine was reacted with 3-chloropropyltrimethoxysilane in anhydrous ethanol. The reaction mixture was heated and stirred in an oil bath and the silane quaternary ammonium salt was obtained as an ethanol solution.

In the third step, SiO_2_ nanoparticles were modified with the silane quaternary ammonium salt. The nanoparticles were dispersed in anhydrous ethanol and then combined with the silane solution. Hydrochloric acid was added to adjust the pH to 4.0 and the mixture was reacted at 80 °C for 5 h. The resulting modified particles were centrifuged, washed with anhydrous ethanol, and dried.

##### Preparation of Carbon Quantum Dots

**Figure 3:**
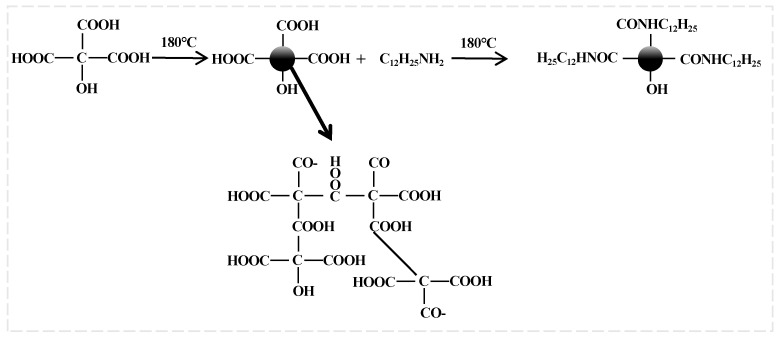
Synthesis route of carbon quantum dots (CQDs).

Carbon quantum dots (abbreviated as “CQDs”; the following will use the abbreviation) were synthesized through a two-step process. The reaction mechanism is illustrated in Figure 3. In the first step, citric acid underwent an esterification reaction under hydrothermal conditions to form carbon particles with a size of a few nanometers [47]. Subsequently, an amide reaction between dodecylamine and the carboxyl groups on the CQDs’ surface introduced long carbon chains, rendering the CQDs hydrophobic.

First, we dispersed citric acid and dodecylamine in anhydrous ethanol, subsequently reacting the mixture at 180 °C in a high-temperature reactor for 3 h. The resulting product was then purified by removing larger particles using a low-speed centrifuge, followed by liquid–liquid extraction with water to separate the desired compound. The final product was obtained as an orange–yellow powder after drying.

##### Assembly of CQDs/Nano-SiO_2_ Nanoparticles

Mesoporous Nano-SiO_2_ exhibits the combined properties of mesoporous materials and nanomaterials, offering controllable morphology, size, and pore structure. These chemically and thermally stable materials possess a mesoporous architecture that provides ample storage space and efficient diffusion pathways for various substances. Consequently, they excel at encapsulating and delivering guest molecules while readily integrating with other nanoparticles. The nanoscale dimensions of these particles facilitate rapid and uniform dispersion of encapsulated molecules.

Carbon quantum dots (CQDs) were synthesized through a hydrothermal process, wherein small carbon-containing molecules aggregated and polymerized into larger structures. By adjusting the hydrothermal conditions, the degree of precursor decomposition could be controlled. This environmentally benign method imposes minimal constraints on carbon source selection. Leveraging the high specific surface area and abundant porosity of mesoporous Nano-SiO_2_, CQDs can be effectively adsorbed, mitigating aggregation issues.

**Figure 4:**
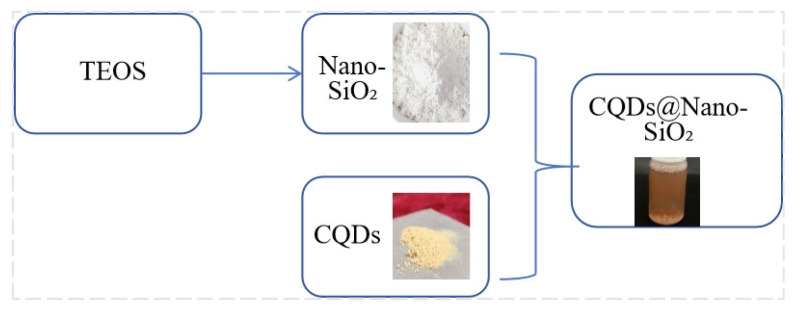
Preparation route of CQDs/Nano-SiO_2_.

To synthesize the CQDs/Nano-SiO_2_ composite material, Nano-SiO_2_ was added to a solution containing CQDs. The mixture was magnetically stirred for 25 min, followed by vacuum drying for 7 h [48]. This process is illustrated in Figure 4.

**Figure 5:**
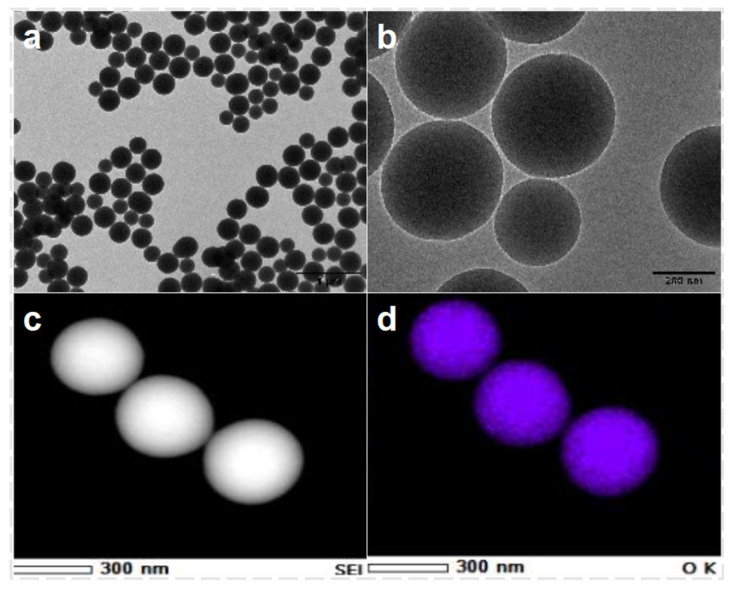
(**a**,**b**) TEM images of CQDs/Nano-SiO_2_; (**c**,**d**) EDS scan of CQDs/Nano-SiO_2_.

The TEM images of CQDs/Nano-SiO_2_ composites are shown in Figure 5. The microspheres prepared by this method are uniform in size and well dispersed. The incorporation of CQDs was confirmed by EDS scans. Figure 5c,d show the C and Si scans of CQDs/Nano-SiO_2_, respectively. The scans in Figure 5a,b clearly show the carbon distribution conforming to the shape of the silica spheres, which is consistent with the distribution of O and Si.

## 3. Compound Experiment

### 3.1. Determination of Foam Drainage Agent Dosage

Using simulated water samples with a salinity of 35 × 10^4^ mg/L, the concentration of foam drainage agent QAFDA is varied and the condensate oil dosage is set at 50 cm^3^/m^3^. The relevant indicators are examined at 80 °C, with an initial liquid volume of 250 mL.

**Figure 6:**
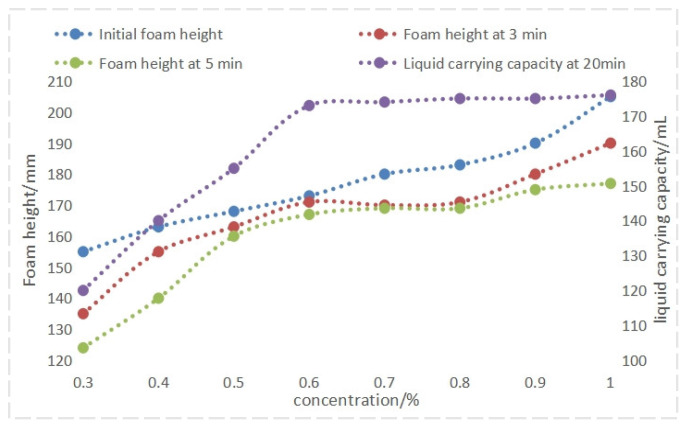
Foam height and liquid holdup at different foam drainage agent concentrations.

**Figure 7:**
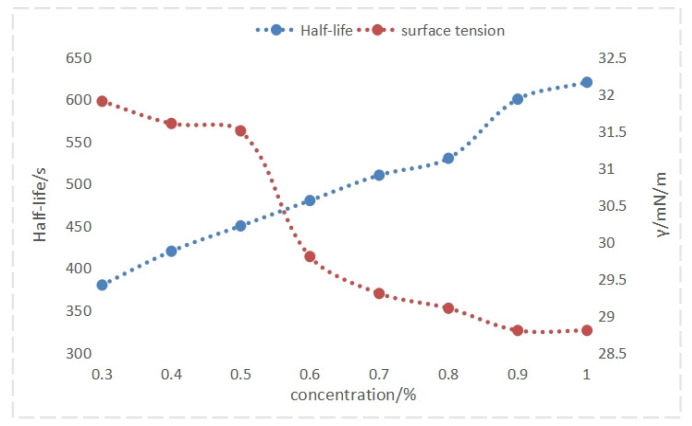
Half-life and surface tension at different foam drainage agent concentrations.

As illustrated in in Figure 6 and Figure 7, increasing the concentration of foam drainage agent QAFDA leads to a decrease in solution surface tension and improved foam performance. Foam performance reaches the desired level at a concentration of 0.5%. While further increasing the concentration to above 0.9% results in minimal changes in surface tension and slight enhancements in foaming and stability, the liquid-carrying capacity remains relatively unchanged. At this point, the concentration has reached the critical micelle concentration. Consequently, an optimal foam drainage agent QAFDA concentration of 0.9% is determined.

### 3.2. Compound Experiment Condition

An orthogonal test design [49,50] was employed to optimize the foam system. Simulated water, prepared using Hechuan gas field water with a salinity of 350,000 mg/L, a condensate oil content of 250 cm^3^/m^3^, and a temperature of 80 °C, served as the experimental medium. The total foam drainage agent concentration was fixed at 0.9% QAFDA, while the mass ratio of QAFDA to CQDs/Nano-SiO_2_ foam stabilizer was varied. Foam performance was evaluated based on initial foam height, foam height after 3 min, and liquid-carrying capacity after 20 min, using an initial liquid volume of 250 mL. 

**Figure 8:**
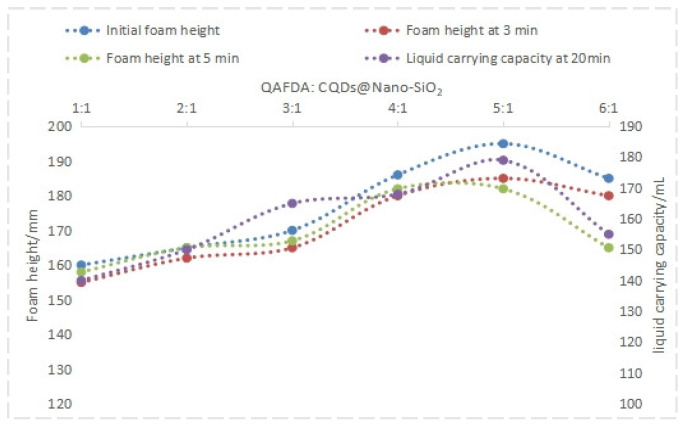
Foam height and liquid holdup under different ratios.

**Figure 9:**
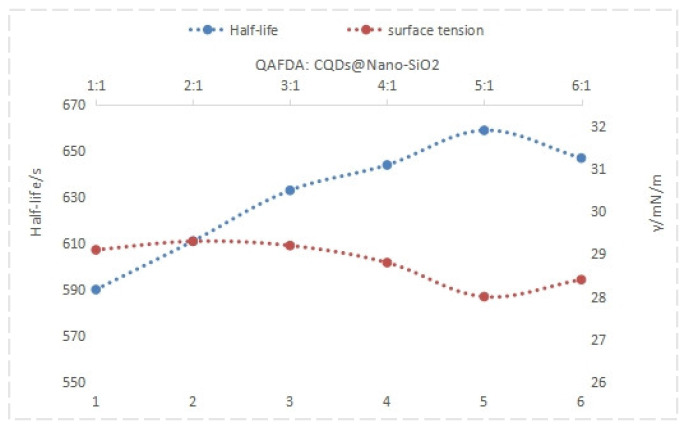
Half-life and surface tension under different ratios.

Analysis of the data in Figure 8 and Figure 9 reveals that the QAFDA-to-CQDs/Nano-SiO_2_ mass ratio significantly influences foam drainage performance. The optimal ratio of 5:1 yielded superior results, characterized by an initial foam height of 195 mm, a foam height of 185 mm after 3 min, a foam height of 182 mm after 5 min, a liquid-carrying capacity of 179 mL, a half-life of 659 s, and a surface tension of 28.1 mN/m.

## 4. Results and Discussion

### 4.1. Performance Evaluation of Foam Drainage System

#### 4.1.1. Evaluation of Temperature Resistance

Prepare a simulated water sample with a mineralization degree of 35 × 10^4^ mg/L. The foam drainage agent composition was fixed at a 5:1 mass ratio of QAFDA to CQDs/Nano-SiO_2_ with a total dosage of 1.1%. Temperature was varied as the experimental parameter, while initial foam height, foam height after 3 min, and liquid-carrying capacity after 20 min were used as performance indicators. An initial liquid volume of 250 mL was maintained throughout the experiments.

**Figure 10:**
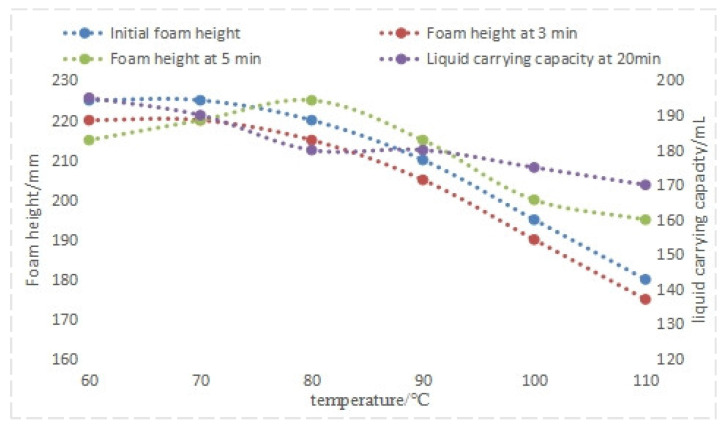
Performance of foam drainage agent at different temperatures.

Analysis of the experimental data in Figure 10 indicates a negative correlation between temperature and foam drainage agent system performance. At 80 °C, the initial foam height, foam height at 3 min and 5 min, and liquid-carrying capacity at 20 min are all at high levels. However, at 90 °C, the performance indicators show a downward trend. Nevertheless, there is a significant improvement compared to using only a single main foam drainage agent without the addition of nanoparticles.

#### 4.1.2. Evaluation of Mineralization Resistance

Simulated water samples with varying salinities were prepared. A fixed QAFDA-to-CQDs/Nano-SiO_2_ mass ratio of 5:1 and a total dosage of 1.1% were maintained throughout the experiment. Foam performance was evaluated at 80 °C, using initial foam height, foam height after 3 min, and liquid-carrying capacity after 20 min as assessment metrics. An initial liquid volume of 250 mL was used for all tests. 

**Figure 11:**
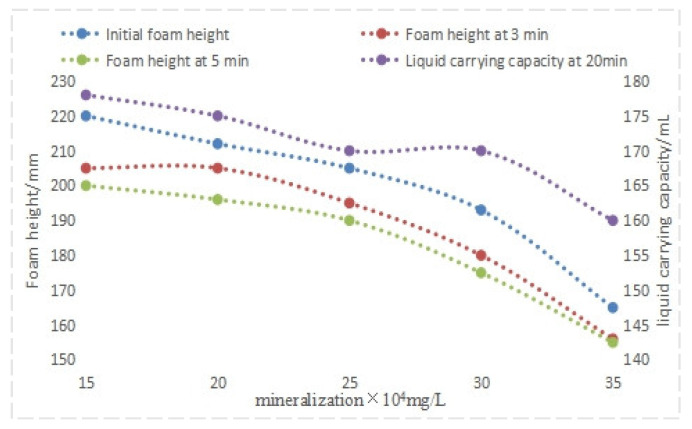
Performance of foam drainage agent at different salinities.

Analysis of Figure 11 suggests that increased temperature diminishes the foaming ability of the agent system while exhibiting a tendency to enhance foam stability. This apparent discrepancy arises from the thermal expansion of the foam drainage agent post-foam generation, initially leading to an exaggerated foam height. However, subsequent foam height reduction and decreased liquid-carrying capacity with rising salinity counteract this effect. Despite these trends, the foam drainage agent system consistently meets performance criteria at salinities up to 350,000 mg/L.

#### 4.1.3. Evaluation of Anti-Condensate Oil Performance

A simulated water sample with a mineralization degree of 35 × 10^4^ mg/L was prepared. A mass ratio of QAFDA to CQDs/Nano-SiO_2_ of 5:1 was selected, with an addition of 1.1%. The amount of condensate oil varied and the initial foam height, foam height after 3 min, and liquid-carrying capacity after 20 min at 80 °C were examined. An initial liquid volume of 250 mL was used.

**Figure 12:**
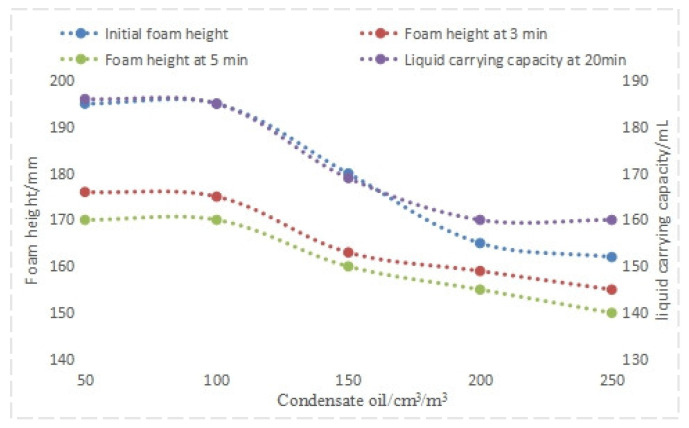
Performance of foam drainage agent with different amounts of condensate oil.

From the analysis of experimental data in Figure 12, as the amount of condensate oil increases, the performance of the foaming agent system is significantly affected. When the amount of condensate oil is within 250 cm^3^/m^3^, the performance indicators of the foaming agent system can meet the requirements.

#### 4.1.4. Effect of Different Aging Times on the Performance of the Foam Drainage Agent System

A simulated water sample was prepared with a mineralization degree of 35 × 10^4^ mg/L. Using a 5:1 mass ratio of QAFDA to CQDs/Nano-SiO_2_ and a total dosage of 1.1%, a 250 mL foam drainage agent solution was prepared at 80 °C. The solution was allowed to age in an oven for varying durations before evaluating initial foam height, foam height after 3 min, and liquid-carrying capacity after 20 min, all at a constant temperature of 80 °C.

**Figure 13:**
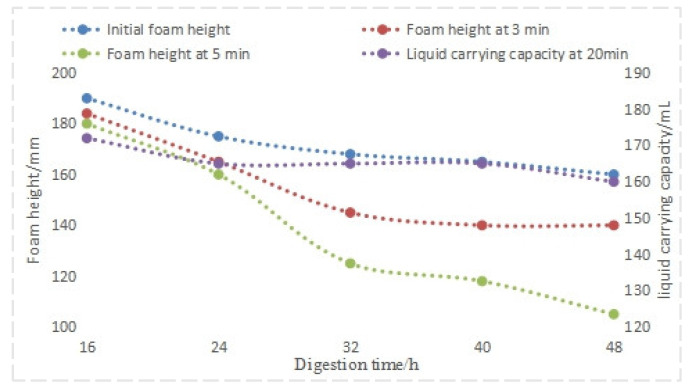
Performance of foam drainage agent at different aging times.

Analysis of the experimental data from Figure 13 suggests that aging time significantly impacts foam drainage agent system performance, particularly with respect to foam stability. While minimal performance changes occur within the first 24 h, foam stability deteriorates rapidly beyond 16 h of aging. Notably, even after 48 h, performance indicators remain within acceptable limits.

### 4.2. Effect of Foam Drainage Agent Concentration on Surface Tension and Half-Life

#### 4.2.1. Effect of Foam Drainage Agent Concentration on Solution Surface Tension

Using simulated water (mineralization degree of 35 × 10^4^ mg/L, condensate oil 250 cm^3^/m^3^), different concentrations of foam drainage agent were added. The changes in surface tension of the solution at temperatures of 25 °C and 80 °C were measured.

**Figure 14:**
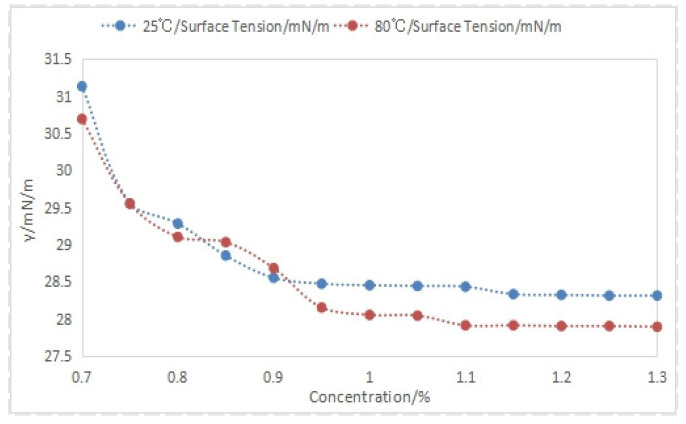
Effect of different foam drainage agent concentrations on surface tension.

Analysis of the experimental data from Figure 14 reveals that increasing foam drainage agent concentration reduces solution surface tension for both formation water and simulated water. However, the magnitude of this reduction is influenced by temperature and solution composition. Higher temperatures correlate with lower surface tension due to increased molecular kinetic energy and weakened intermolecular forces. Additionally, the presence of condensate oil in simulated water decreases surface tension compared to formation water at equivalent conditions. Notably, a foam drainage agent concentration of 1.1% stabilizes surface tension.

#### 4.2.2. Effect of Concentration on the Half-Life of Foam Drainage Agent

Employing both formation water (mineralization: 298,400 mg/L) and simulated water (mineralization: 350,000 mg/L; condensate oil: 250 cm^3^/m^3^), the half-life of foam generated by various foam drainage agent concentrations was measured at a constant temperature of 80 °C.

**Figure 15:**
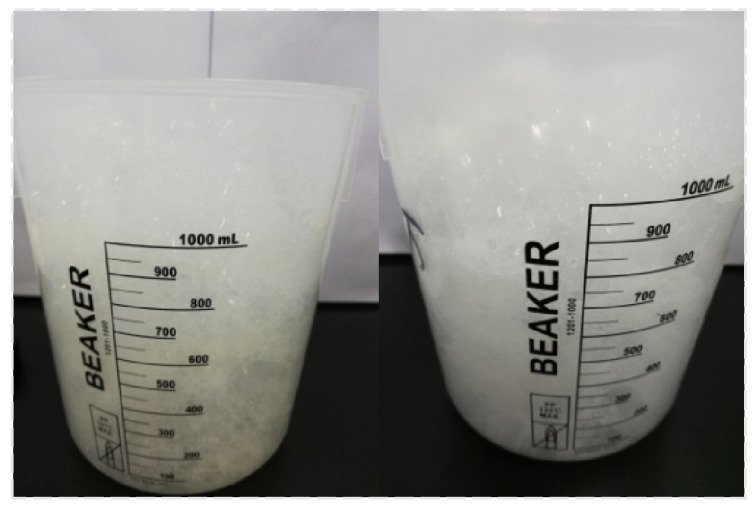
Comparison of half-life of foam drainage system concentrations of 1% and 1.1% in formation water.

**Figure 16:**
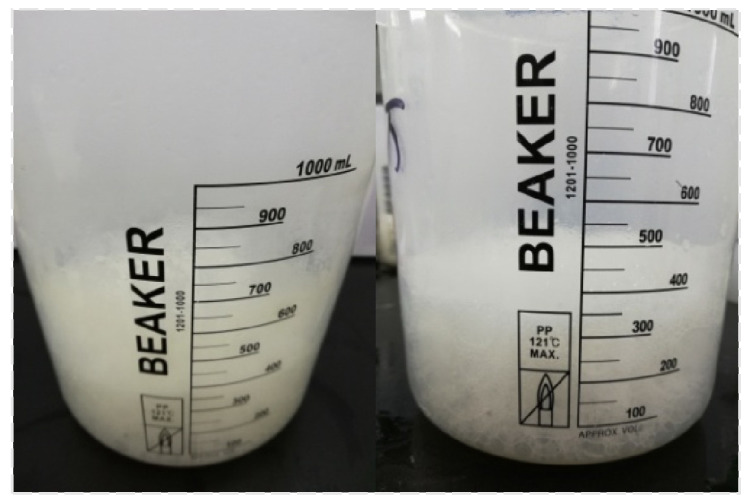
Comparison of half-life of foam drainage system concentrations of 1% and 1.1% in simulated water.

Analysis of the experimental data presented in Table 1 and Figure 15 and Figure 16 reveals that foam generated from water samples containing condensate oil exhibits greater density, stability, and a longer half-life compared to foam produced from formation water. These findings demonstrate the foam drainage agent system’s robust resistance to both high mineralization and condensate oil contamination.

## 5. Conclusions

This study developed a novel, high-performance foam drainage system, containing nanoparticles, tailored to the challenging conditions prevalent in the Hechuan gas field. Characterized by high salinity, significant temperature variations, and substantial condensate oil content, this environment demanded a specialized foam drainage agent. To address these challenges, a quaternary ammonium foam drainage agent was synthesized, exhibiting exceptional resistance to both high salinity and condensate oil. To further enhance foam stability and performance, carbon quantum dots/silica nanoparticles were prepared. Through systematic optimization, a foam drainage system combining quaternary ammonium foam drainage agent and carbon quantum dots and silica nanoparticles as the stabilizer was developed. This system demonstrated superior performance under extreme conditions, including high salinity, elevated temperature, and the presence of condensate oil. Key performance indicators such as foam height, stability, and liquid-carrying capacity met or exceeded the requirements for effective gas well foam drainage in the Hechuan gas field.

The successful development of this novel foam drainage system highlights the efficacy of nanoparticle-enhanced foam drainage agent in overcoming the challenges posed by complex and harsh reservoir environments. This foam drainage system can be applied to gas fields with a salinity of 35 × 10^4^ mg/L, filling the gap where most high-salinity gas fields had no suitable agents available. This research provides a valuable foundation for the design and development of advanced foam systems for future applications in the oil and gas industry.

While the foam drainage agents developed in this study demonstrate promising applications in high-salinity gas fields, their adaptability may gradually decline as gas field development progresses and understanding deepens. Therefore, ongoing research and improvements are essential to ensure their continued effectiveness. Given the systemic nature of gas field operations, the impact of a single well or process is limited. Comprehensive water control research at the reservoir level is crucial for economically and efficiently maintaining stable production across the entire field and maximizing ultimate recovery. Moreover, the integration of big data technology can enhance intelligent control and performance evaluation. With the growing number of low-yield and low-pressure gas wells, the adaptability and economic efficiency of existing drainage gas recovery processes face significant challenges. Future research should focus on developing innovative technologies and strategies to address these challenges and ensure the sustainable production of gas from unconventional reservoirs.

## Figures and Tables

**Table 1:** Effect of different foam drainage agent concentrations on surface tension.

Concentration/%	Formation Water (29.84 × 10^4^ mg/L)	Simulated Water (35 × 10^4^ mg/L)
1.0	725	632
1.1	789	659

## Data Availability

The original contributions presented in this study are included in the article; further inquiries can be directed to the corresponding author.

## Abbreviations

Serial number	Abbreviated form	Full English name
1	CQDs	Carbon Quantum Dots
2	CQDs/Nano-SiO_2_	Carbon Quantum Dots/Silica Nanoparticles
3	Nano-SiO_2_	Silica Nanoparticles
4	QAFDA	Quaternary Ammonium Foam Drainage Agent
5	TEOS	Tetraethyl Orthosilicate
